# *Burkholderia mallei* and *Burkholderia pseudomallei* Cluster 1 Type VI Secretion System Gene Expression Is Negatively Regulated by Iron and Zinc

**DOI:** 10.1371/journal.pone.0076767

**Published:** 2013-10-11

**Authors:** Mary N. Burtnick, Paul J. Brett

**Affiliations:** Department of Microbiology and Immunology, University of South Alabama, Mobile, Alabama, United States of America; University of Toledo School of Medicine, United States of America

## Abstract

*Burkholderia mallei* is a facultative intracellular pathogen that causes glanders in humans and animals. Previous studies have demonstrated that the cluster 1 type VI secretion system (T6SS-1) expressed by this organism is essential for virulence in hamsters and is positively regulated by the VirAG two-component system. Recently, we have shown that T6SS-1 gene expression is up-regulated following internalization of this pathogen into phagocytic cells and that this system promotes multinucleated giant cell formation in infected tissue culture monolayers. In the present study, we further investigated the complex regulation of this important virulence factor. To assess T6SS-1 expression, *B. mallei* strains were cultured in various media conditions and Hcp1 production was analyzed by Western immunoblotting. Transcript levels of several VirAG-regulated genes (*bimA*, *tssA*, *hcp1* and *tssM*) were also determined using quantitative real time PCR. Consistent with previous observations, T6SS-1 was not expressed during growth of *B. mallei* in rich media. Curiously, growth of the organism in minimal media (M9G) or minimal media plus casamino acids (M9CG) facilitated robust expression of T6SS-1 genes whereas growth in minimal media plus tryptone (M9TG) did not. Investigation of this phenomenon confirmed a regulatory role for VirAG in this process. Additionally, T6SS-1 gene expression was significantly down-regulated by the addition of iron and zinc to M9CG. Other genes under the control of VirAG did not appear to be as tightly regulated by these divalent metals. Similar results were observed for *B. pseudomallei*, but not for *B. thailandensis*. Collectively, our findings indicate that in addition to being positively regulated by VirAG, *B. mallei* and *B. pseudomallei* T6SS-1 gene expression is negatively regulated by iron and zinc.

## Introduction

*Burkholderia mallei*, the etiologic agent of glanders, is a Gram-negative bacillus that primarily infects horses, mules and donkeys, and occasionally humans. In nature, chronically infected horses are believed to be the only reservoir of this host-adapted pathogen [1]–[4]. Although glanders has been eradicated from most countries, it remains endemic in parts of Central and South America, the Middle East, Africa, and Asia, and has gained re-emerging status due to several recent outbreaks in these areas [2], [5]–[10]. Disease in equines presents as chronic or acute illnesses that are commonly characterized by the presence of caseous pulmonary nodules, nasal ulcerations and cutaneous abscess formation [4], [8], [11]. Natural human infections are acquired via the inoculation of mucocutaneous tissues with aerosols or secretions from diseased animals. The clinical progression of human glanders is similar to that observed in equines and acute disease is frequently fatal if not promptly recognized and treated [12], [13]. At present, there are no human or veterinary vaccines available for immunization against *B. mallei*. Due to the high risk of aerosol infection and the historical use of this organism as a biological weapon, *B. mallei* is currently listed as a Tier 1 Select Agent by the Centers for Disease Control and Prevention (CDC) [4], [12], [14], [15].

*B. mallei* is a facultative intracellular pathogen that can survive and replicate within the cytosol of eukaryotic cells. Several studies have shown that the organism persists within murine macrophage cell lines *in vitro* and can be observed within phagocytic cells in animal models of glanders [11], [16]–[21]. Following uptake by host cells, *B. mallei* rapidly escapes from endocytic vacuoles, enters into the cytosol and uses actin-based motility to promote intra- and inter-cellular spread [16], [19], [21]. *B. mallei* also possesses the unique ability to induce host cell fusion resulting in multinucleated giant cell (MNGC) formation; a phenomenon that is observed both *in vitro* and *in vivo* [16], [19], [20]. Several major virulence factors that are required for survival of *B. mallei* in animals have recently been shown to influence key processes during the interactions of the organism with eukaryotic cells. Included among these are an animal pathogen-like Type III secretion system (T3SS_AP_), the cluster 1 Type VI secretion system (T6SS-1) and the VirAG two-component regulatory system [17], [18], [21]–[23]. While T3SS_AP_ is essential for early vacuolar escape, T6SS-1 is necessary for optimal intracellular growth, robust actin-based motility, and MNGC formation in RAW 264.7 murine macrophages [17], [18], [21]. In addition, *virG* is required for actin tail formation by *B. mallei* in J774.2 cells [23]. At present, the molecular mechanisms underlying the altered phenotypes associated with *B. mallei* T3SS_AP,_ T6SS-1 and VirAG mutants are unclear.

Type VI secretion systems (T6SSs) are contact-dependent protein translocation machines expressed by many Gram-negative bacteria [24]–[28]. Mounting evidence indicates that these systems resemble inverted bacteriophage-like puncturing devices that deliver bacterial effector proteins directly into eukaryotic and/or prokaryotic target cells [24], [29]–[39]. Two hallmark proteins associated with most T6SSs are hemolysin co-regulated protein (Hcp) and valine-glycine repeat protein (VgrG) [27], [28]. While Hcp and VgrG are integral components of the T6SS apparatus, both can also be detected in the culture supernatants of bacteria having functional T6SSs [24], [27], [29], [40]. In addition, some “evolved” VgrGs have been identified and shown to contain effector functions (e.g. actin cross-linking activity) in their C-terminal extended regions [27], [29], [41]. Recently, a few non-VgrG, T6SS-secreted proteins have been identified and shown to exhibit anti-bacterial activities [27], [42]–[45]. With the exception of the *Vibrio cholerae* VasX protein, T6SS-secreted, non-VgrG effectors that act directly on eukaryotic targets remain largely unidentified [46].

T6SS expression is precisely regulated at the genetic level and typically involves two-component regulatory systems, transcriptional activator proteins, posttranscriptional modulators or posttranslational activation mechanisms [28], [47], [48]. At present, the specific signals that modulate T6SS gene expression and the environmental cues that influence these pathways are only beginning to be identified [28], [47], [48]. In a variety of pathogens, T6SSs are expressed following interactions with tissue culture or with host cells *in vivo*, but are repressed during routine growth in bacteriological culture media [17], [23], [48]–[57]. Studies from our laboratory and others have shown that *B. mallei* T6SS-1 genes are transcribed poorly during the routine culture in rich medium, but are expressed following uptake by murine macrophages prior to vacuolar escape [17], [23]. In addition, T6SS-1 gene expression is controlled, at least in part, by VirAG and the AraC-type regulator BMAA1517 [23]. Mass spectroscopy studies have demonstrated that when either of these regulators is overexpressed during *in vitro* growth of *B. mallei*, Hcp1 becomes the major protein detected in culture supernatants [23]. Currently, the specific environmental cue(s) that lead to T6SS-1 gene expression are unknown.

In the present study, we sought to identify defined growth conditions that would induce T6SS-1 gene expression *in vitro* while still supporting robust growth of *B. mallei*. We describe the use of minimal media formulations that facilitate Hcp1 production and demonstrate that *virG* is required for T6SS-1 gene expression during growth in this media. In addition, we show that divalent metals influence T6SS-1 gene expression and Hcp1 production. Since two closely related species, *B. pseudomallei* (etiologic agent of melioidosis) and *B. thailandensis* (non-pathogenic environmental saprophyte), also harbor homologous T6SS-1 gene clusters in their genomes [23], we extended our studies to examine Hcp1 production by these organisms during growth under our defined media conditions.

## Materials and Methods

### Bacterial Strains, Growth Conditions and Reagents

The bacterial strains used in this study are shown in Table 1. *Escherichia coli*, *B. pseudomallei* and *B. thailandensis* strains were routinely cultured at 37°C on Luria Bertani (LB) agar (Lennox L agar; Difco) or in LB broth (Lennox L broth, Difco). *B. mallei* strains were routinely cultured at 37°C on LB agar or in LB broth supplemented with 4% glycerol (LB4G). For growth of the *Burkholderia* strains in minimal media, the following formulations were used: Difco M9 Minimal Salts supplemented with 0.4% glucose (M9G); M9G supplemented with 0.5% (w/v) Bacto Casamino Acids (M9CG); or M9G supplemented with 0.5% Bacto Tryptone (M9TG). Metal-deplete M9TG media (M9TG-C) was prepared by supplementing M9G media with 0.5% Bacto Tryptone that had been chelated two times with 2.5% (w/v) Chelex 100. When appropriate, antibiotics were added at the following concentrations: 25 µg/ml kanamycin (Km), 50 µg/ml zeocin (Zeo), 15 µg/ml polymyxin B (Pm), and 100 µg/ml ampicillin (Ap) for *E. coli*; 5 µg/ml Km, 5 µg/ml Zeo for *B. mallei*; and 100 µg/ml Km for *B. thailandensis*. Bacterial stocks were maintained at −80°C as 20% glycerol suspensions. All studies utilizing viable *B. mallei* and *B. pseudomallei* were conducted in a CDC select agent-certified biosafety level 3 containment facility.

**Table 1:** Strains, plasmids and PCR primers used in this study.

Strain, plasmid or primer	Relevant characteristics	Reference
***E. coli***		
TOP10	General cloning strain: Ap^S^, Km^S^, Zeo^S^	Invitrogen
S17-1	Mobilizing strain; transfer genes of RP4 integrated on chromosome: Pm^S^, Km^S^, Zeo^S^	[70]
***B. mallei***		
ATCC 23344	Type strain; human isolate from China: Pm^R^, Km^S^, Zeo^S^	[22]
NCTC 3708	Mule isolate from India	[71]
NCTC 3709	Horse isolate from India	[71]
SR1A	ATCC 23344 derivative; sucrose-resistant: Pm^R^, Km^S^, Zeo^S^	[17]
BM0742	SR1A derivative; Δ*hcp1*: Pm^R^, Km^S^	This study
BM0746	SR1A derivative; Δ*virG*: Pm^R^, Km^S^, Zeo^S^	This study
***B. pseudomallei***		
K96243	Human isolate from Thailand	[72]
1026b	Human isolate from Thailand	[73]
***B. thailandensis***		
E264	Type strain (ATCC 700388); environmental isolate from Thailand	[74]
DW503	E264 derivative; Δ(*amrR-oprA*): Pm^R^, Km^S^	[75]
**Plasmids**		
pEX18Zeo	Gene replacement vector; *sacB*, *oriT*: Zeo^R^	[17]
pEXZΔvirG	pEX18Zeo containing *virG* with an internal 210 bp deletion: Zeo^R^	This study
pMo130-ΔBPSS1498	pMo130 derivative containing *hcp1* with an internal 126 bp deletion: Km^R^	[50]
pBHR2	Broad host range cloning vector; pBBR1 *oriR*, *oriT*: Km^R^	[23]
pvirAG	pBHR2 containing a wild type copy of *B. mallei virAG* (aka pBHR2*-virAG)*: Km^R^	[23]
pBtvirAG	pBHR2 containing a wild type copy of *B. thailandensis virAG*: Km^R^	This study
pBAD/HisA	Arabinose inducible expression vector; pBR322 *ori*: Ap^R^	Invitrogen
pBmhcp1-His	BmHcp1-6xHis expression construct; pBAD/HisA derivative: Ap^R^	This study
pBthcp1-His	BtHcp1-6xHis expression construct; pBAD/HisA derivative: Ap^R^	This study
**PCR Primers** a		
BPSS1498-1	5′-GCTAGCATCCGCCAGTACGTCGTCGAC-3′	[50]
BPSS1498-4	5′- GCTAGCTCAGGAAATCGTTCGGATATC-3′	[50]
virG-RH	5′-CATG*AAGCTT*AATGACGTTCGACGAGATCG-3′	This study
virG-FKp	5′-CATG*GGTACC*TCTTCGAGCCGTTCTATTCG-3′	This study
BtvirG-FKp	5′-CATG*GGTACC*AGCCGCTTCAGGCTCACGTTGC-3′	This study
BtvirAG2-RXb	5′-CATG*TCTAGA*TCGAAATCCTCGGGAAGAAGCGCAC-3′	This study
Bmhcp1-HisF1	5′-CCCAAC*GGTCTCA*CATGCTGGCCGGAATATATCTCAAGG-3′	This study
Bmhcp1-HisR1	5′-CCCAAC*GGTCTCA*AGCTTCAATGATGATGATGATGATGCGCCGCCGCGCCATTCGTCCAGTTTGCGGC-3′	This study
Bthcp1-HisF1	5′-CCCAAC*GGTCTCA*CATGCCGATGCCGTGCTATCTCACGC-3′	This study
Bthcp1-HisR1	5′-CCCAAC*GGTCTCA*AGCTTCAATGATGATGATGATGATGCGCCGCCGCCACCTTCGGCGCGCGCCATGA-3′	This study

a Restriction sites in the linker regions are italicized.

Unless stated otherwise, chemicals were purchased from Sigma-Aldrich. Zeocin was purchased from Invitrogen. Puratronic metals were obtained from Alfa Aesar as follows: Cu as Copper (II) sulfate, 99.999%; Fe as Iron (II) sulfate, 99.999%; Mg as Magnesium sulfate, 99.997%; Mn as Manganese (II) sulfate, 99.999%; Ni as Nickel (II) sulfate, 99.9985%; Zinc sulfate, 99.999%.

### Recombinant DNA Techniques

DNA manipulations were performed using standard methods. Restriction enzymes and T4 DNA Ligase (New England Biolabs) were used according to manufacturer’s instructions. PCR was performed using an Expand High Fidelity PCR System (Roche Applied Science) or GoTaq DNA Polymerase (Promega); 1 M Betaine was included in all polymerase chain reaction (PCR) procedures. PCR was performed using the following conditions: 97°C for 5 min; 30 cycles, each consisting of 97°C for 45 s, 55°C for 45 s, and 72°C for 3 min; a final extension step of 72°C for 10 min was included. PCR and restriction digested products were purified using a QIAquick Gel Extraction Kit (Qiagen). Plasmids were purified using a QIAprep Spin Miniprep Kit (Qiagen). Genomic DNA was purified using a Wizard Genomic DNA Purification kit (Promega). Chemically competent *E. coli* TOP10 cells were transformed as per manufacturer’s instructions (Invitrogen). Oligonucleotide primers and dual-labeled probes were obtained from Integrated DNA Technologies or Sigma-Aldrich. DNA sequencing was performed by ACGT Inc. The plasmids and primers used in this study are shown in Table 1.

### Mutant Construction and Plasmid Conjugations

Gene replacement experiments with *B. mallei* SR1A were performed with the *sacB*-based allelic exchange vectors pEX18Zeo and pMo130 [17], [23], [58]. To construct pEXZΔvirG, the virG-FH/virG-RKp primer pair was used to PCR amplify the Δ*virG* gene (210-bp markerless, in-frame deletion of the *virG* gene) from pGRV2-ΔA0746 [23]. The PCR product was then digested with *Hind*III and *Kpn*I and cloned into pEX18Zeo digested with the same enzymes resulting in plasmid pEXZΔvirG. *E. coli* S17-1 was used to mobilize pEXZΔvirG or pMo130-ΔBPSS1498 into *B. mallei* SR1A via conjugative mating essentially as previously described [17], [50]. Briefly, overnight cultures of S17-1 (pEXZΔvirG or pMo130-ΔBPSS1498) and SR1A were pelleted by centrifugation, resuspended together in 100 µl of 10 mM MgSO_4_, spotted onto LB4G agar plates and incubated for 8 h at 37°C. To select for transconjugants, mating mixtures were plated onto LB4G-Pm-Zeo or LB-Pm-Km agar and incubated at 37°C for 48 h. To select for sucrose resistant colonies, transconjugants were inoculated into LB4G broth without antibiotics and 10-fold dilutions of the overnight cultures were spread onto M9G plates containing 5% sucrose. Following incubation at 37°C for 48–72 h, sucrose resistant colonies were screened for loss of the Zeo or Km resistance markers by replica plating individual colonies onto LB4G and LB4G-Zeo or LB4G-Km. The resolved co-integrates were screened for the presence of the mutant alleles (Δ*virG* or Δ*hcp1*) by PCR using the appropriate primer pairs (virG-FH/virG-RKp or BPSS1498-1/BPSS1498-4).

For *virAG* expression experiments, pBHR2 and pvirAG were mobilized into *B. mallei* SR1A and its derivatives via conjugative mating as described above and transconjugants were selected for on LB4G-Pm-Km agar. Plasmid pBtvirAG was constructed by PCR amplifying *virAG* from *B. thailandensis* E264 genomic DNA using the BtvirAG-FKp/BtvirAG2-RXb primer pair and then cloning the resulting PCR product into the *Kpn*I and *Xba*I sites of pBHR2. pBtvirAG was mobilized into *B. thailandensis* DW503 via conjugative mating essentially as described for *B. mallei*.

### Hcp1 Expression, Purification and Antibody Production

For expression of recombinant *B. mallei* Hcp1 with a C-terminal 6xHis-Tag (BmHcp1-6xHis), the *hcp1* ORF (BMAA0742) was PCR amplified from *B. mallei* ATCC 23344 genomic DNA using the Bmhcp1-HisF1 and Bmhcp1-HisR1 primer pair. For expression of recombinant *B. thailandensis* Hcp1 with a C-terminal 6xHis-Tag (BtHcp1-6xHis), the *hcp1* ORF (BTH_II0868) was PCR amplified from *B. thailandensis* E264 genomic DNA using the Bthcp1-HisF1 and Bthcp1-HisR1 primer pair. The resulting DNA fragments were digested with *Bsa*I and cloned into pBAD/HisA digested with *Nco*I/*Hind*III resulting in pBmhcp1-His and pBthcp1-His, respectively.

For purification of BmHcp1-6xHis or BtHcp1-6xHis, *E. coli* TOP10 (pBmhcp1-His) or TOP10 (pBthcp1-His) were grown in 500 ml of LB broth plus Ap at 37°C with aeration (200 rpm). When the cultures reached an OD_600_ of 0.8, protein expression was induced using 0.02% L-arabinose for 5 h. Bacteria were harvested by centrifugation (8000×g, 10 min, 4°C), resuspended in B-PER (Pierce) plus 1 µl/ml Benzonase (Novagen) and incubated for 10 min at room temperature (RT). The insoluble material was pelleted by centrifugation (17000×g, 15 min, 4°C) and resuspended in B-PER and incubated for 10 min at RT. The insoluble material was again pelleted, then resuspended in Solubilization Buffer (50 mM Tris (pH 8.0), 50 mM NaCl, 10 mM Imidazole and 0.2% Sarkosyl) and gently agitated for 60 min at RT. The remaining insoluble material was removed by centrifugation, the supernatant was filter sterilized and loaded onto a gravity fed Ni-NTA agarose (Qiagen) column. The flow through was collected and applied to the column a second time followed by washing of the column with Wash Buffer (50 mM Tris (pH 8.0), 300 mM NaCl and 40 mM Imidazole). Protein was eluted with Elution Buffer (50 mM Tris pH 8.0, 50 mM NaCl and 300 mM Imidazole). Fractions were analyzed by SDS-PAGE and those containing BmHcp1-6xHis or BtHcp1-6xHis were pooled, concentrated, dialyzed against saline and stored at −20°C. Protein concentrations were determined using a BCA protein assay kit (Pierce).

Purified BmHcp1-6xHis was used to raise BmHcp1-specific polyclonal antiserum in rats, and purified BtHcp1-6xHis was used to raise BtHcp1-specific polyclonal antiserum in rabbits, at Cocalico Biologicals, Inc. (Reamstown, PA) using a standard protocol.

### Ethics Statement

All procedures involving animals were performed in strict accordance with the recommendations in the Guide for the Care and Use of Laboratory Animals of the National Institutes of Health. All protocols were approved by the Cocalico Biologicals, Inc. Animal Care and Use Committee.

### SDS-PAGE and Western Immunoblotting

Whole cell lysates were prepared from *B. mallei*, *B. pseudomallei* or *B. thailandensis* strains grown in various media (LB, LB4G, M9G, M9CG, M9TG, and M9TG-C or M9CG supplemented with divalent metals) overnight (16–18 h) at 37°C with shaking (200 rpm). Briefly, 0.5–1 ml of bacterial culture was pelleted by centrifugation, the resulting pellets were resuspended in 0.2 ml of 1X Novex Tris-glycine SDS Sample Buffer (Invitrogen) with β-mercaptoethanol and then boiled for 10 min. For immunoblot analyses, the whole cell lysates (10 µl/lane) were separated on 12% Novex Tris-glycine gels (Invitrogen) and electrophoretically transferred to nitrocellulose membranes. The membranes were blocked with StartingBlock T-20 Blocking Buffer (Thermo Scientific) for 20 min, incubated with a polyclonal rat anti-BmHcp1 or rabbit anti-BtHcp1 serum (1/3000 dilution in Tris Buffered Saline containing 0.05% Tween 20; TBS-T) for 1 h, and then incubated with either goat anti-rat or anti-rabbit IgG horse radish peroxidase conjugate antibodies (1/5000 dilution in TBS-T) for 1 h. Membranes were washed three times with TBS-T following each incubation step. Blots were visualized using Pierce ECL Western Blotting Substrate (Thermo Scientific) and a ChemiDoc XRS imaging system (BioRad).

### Quantitative Real-time PCR (qRT-PCR)

For quantitation of transcript levels, RNA was purified from *B. mallei* or *B. pseudomallei* strains grown in various media (LB, LB4G, M9G, M9CG, and M9TG or M9CG supplemented with divalent metals) for 8 h at 37°C with shaking (200 rpm). RNA extracted from bacterial pellets using TRIzol Reagent (Invitrogen) was treated with RNase-free DNase I and purified using an RNeasy Miniprep Kit (Qiagen) according to the manufacturer’s instructions. SuperScript III (Invitrogen) was used to synthesize first-strand cDNA from 1 µg of RNA following manufacturer’s instructions. qRT-PCR primers and probes specific for target genes were designed using Primer Express 3.0 and are shown in Table 2. Reactions were prepared in a total volume of 20 µl using TaqMan Gene Expression Master Mix (Applied Biosystems), 1–2 ng of first-strand cDNA, 300 nM forward and reverse primers, and 250 nM probe. qRT-PCR was performed using a StepOne Real-Time PCR System (Applied BioSystems) under the following conditions: 2 min at 50°C, 10 min at 95°C, followed by 40 cycles at 95°C for 15 s and 60°C for 1 min. Three individual assays were performed in triplicate. Each transcript was normalized by comparison with the constant, internal control *rpoA*. Fold changes were calculated using the ΔΔC_T_ method. The housekeeping gene *dnaK* was used as a control for comparative purposes since expression of this gene has been shown to remain similar in various growth conditions [51]. The data was plotted and analyzed using StepOne Software v2.2.2 (Applied Biosystems) and GraphPad Prism 5 (GraphPad Software Inc.).

**Table 2:** qRT-PCR primers and probes used in this study.

Primer or probe	Sequence^a^	Reference
BmrpoA-RTF1	5′-AAGCTCGTGATGAACATCGAAA-3′	This study
BmrpoA-RTR1	5′-GACAGCTGGTCGACGAGGAT-3′	This study
BmrpoA-RTP1	5′-[6FAM]-CATCACGCCGGAAGAAGCGATCC-[BHQ1]-3′	This study
BmdnaK-RTF1	5′-GCGTGATGACGAAGATGATCA-3′	This study
BmdnaK-RTR1	5′-CGCCCTGGAACACCTTGAT-3′	This study
BmdnaK-RTP1	5′-[6FAM]-CCGACGAAGCACGCTCAGGTGTATTC-[BHQ1]-3′	This study
Bmhcp1-RTF1	5′-GCGCGAGATGATGGAAGAGT-3′	This study
Bmhcp1-RTR1	5′-AGGTCCCCACCTGTTGTATCC-3′	This study
Bmhcp1-RTP1	5′-[6FAM]-CGAGATCACGATCCACCGTCCGA-[BHQ1]-3′	This study
BmtssA-RTF1	5′-TTCGATTCGGTGCACGATT-3′	This study
BmtssA-RTR1	5′-TTGCCGATCGGGCTCTT-3′	This study
BmtssA-RTP1	5′-[6FAM]-TGCCCGAGCTCAAGCAGCTGATC-[BHQ1]-3′	This study
BmvirG-RTF1	5′-CCGCTTCGACGGATGGA-3′	This study
BmvirG-RTR1	5′-AGAAAGAACGCGAGCAGGTT-3′	This study
BmvirG-RTP1	5′-[6FAM]-CCGCTGACGGTCGGCGAATTC -[BHQ1]-3′	This study
BmtssM-RTF1	5′-CGGGATGGAATCTGGTATCG-3′	This study
BmtssM-RTR1	5′-CGGCAGGGCAAGCAAGT-3′	This study
BmtssM-RTP1	5′-[6FAM]-CCAGGTCAGCGCCGTCAACGA-[BHQ1]-3′	This study
BmbimA-RTF1	5′-TTCGCGCATCTACTATGTTCGT-3′	This study
BmbimA-RTR1	5′-AGCGCATCCGTGGAAAAG-3′	This study
BmbimA-RTP1	5′-[6FAM]-ATCGCTCCGCTGGCCCTCG-[BHQ1]-3′	This study
BpbimA-RTF1	5′-CTGCTGAAAACGCTCAATCG-3′	This study
BpbimA-RTR1	5′-GTTGTCGACTACGTCCTCGGTTA-3′	This study
BpbimA-RTP1	5′-[6FAM]-TGTCCGCGGAGCTTCAGAACAACC-[BHQ1] -3′	This study

a [6FAM] = 6-carboxyfluorescein, [BHQ1] = Black Hole Quencher-1.

## Results

### Hcp1 is Produced during Growth of *B. mallei* in Minimal Media

**Figure 1:**
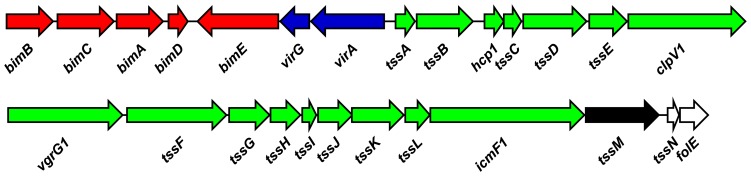
Physical map of the *B. mallei* T6SS-1 gene cluster. Genes encoding components of T6SS-1 are shown in green (BMAA0744-0730). The *Burkholderia* intracellular motility (*bim*) genes are shown in red (BMAA0751-0747), the VirAG two-component regulatory system is shown in blue (*virAG*; BMAA0746-0745), the deubiquitinase encoding gene (*tssM*; BMAA0729) is shown in black, and downstream genes *tssN* and *folE* are shown in white. Homologous gene clusters are also present in *B. pseudomallei* (BPSS1490-1514) and *B. thailandensis* (BTH_II0877-0854).

**Figure 2:**
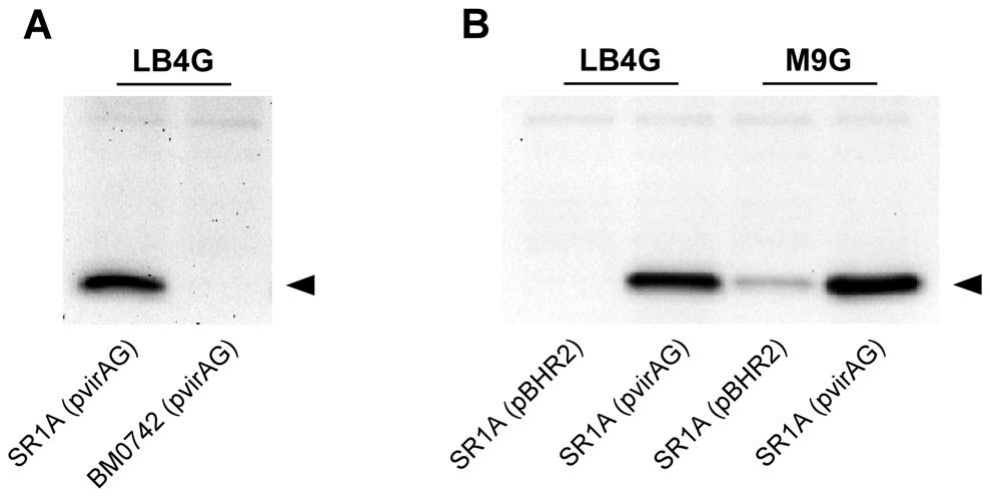
Hcp1 is produced during growth of *B. mallei* in minimal media. Whole cell lysates were prepared from overnight cultures of (A) *B. mallei* SR1A (pvirAG) and BM0742 (pvirAG) grown in LB4G, or (B) *B. mallei* SR1A (pBHR2) and SR1A (pvirAG) grown in LB4G or M9G, and then assayed for Hcp1 production by Western immunoblotting using anti-BmHcp1 polyclonal rat serum. The protein band corresponding to Hcp1 is indicated by the black arrowhead.

The *B. mallei* T6SS-1 gene cluster (BMAA0744-0730) is part of the VirAG regulon and is co-regulated with the *Burkholderia* intracellular motility (*bim*) locus and the clan CA cysteine protease encoding gene, *tssM* (Figure 1) [23]. Previous studies have shown that T6SS-1 genes are not expressed during growth of *B. mallei* in rich media such as LB4G unless *virAG* is expressed from a multicopy plasmid [23]. The goal of the current study was to define media conditions that would support T6SS-1 expression in the absence of plasmid-based expression of *virAG*. To facilitate these studies, we first raised anti-serum against recombinant *B. mallei* Hcp1 (rBmHcp1) in order to monitor T6SS-1 expression during growth of the organism in various media. Utilizing whole cell lysates prepared from *B. mallei* SR1A (pvirAG) and BM0742 (pvirAG), Western immunoblot analysis confirmed the specificity of the polyclonal antiserum for Hcp1 (Figure 2A). Next, we examined Hcp1 production in *B. mallei* SR1A harboring pBHR2 (vector only control) or pvirAG (expresses *virAG*) grown in M9 minimal media formulations. Consistent with previous findings, Western immunoblot analysis confirmed that SR1A (pBHR2) grown in LB4G medium did not produce Hcp1 (Figure 2B). Interestingly, however, Hcp1 was detected when SR1A was grown in M9G media in both the vector only control and the *virAG* expressing strains (Figure 2B). While Hcp1 production was noticeably enhanced when *virAG* was over-expressed, our results suggested that we had identified growth conditions that would enable T6SS-1 expression without the need for plasmid-based expression of *virAG*.

**Figure 3:**
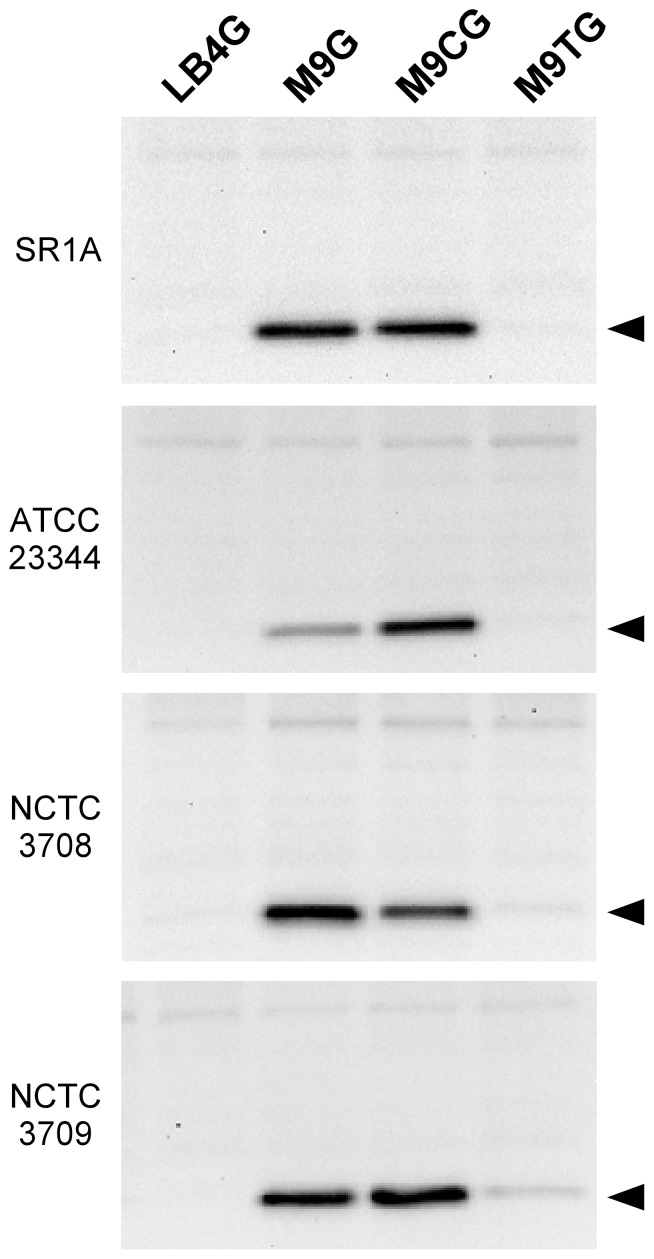
Minimal media supplements influence *B. mallei* Hcp1 production. Whole cell lysates were prepared from overnight cultures of *B. mallei* SR1A, ATCC 23344, NCTC 3708 and NCTC 3709 grown in LB4G, M9G, M9CG or M9TG, and then assayed for Hcp1 production by Western immunoblotting using anti-BmHcp1 polyclonal rat serum. The protein band corresponding to Hcp1 is indicated by the black arrowhead.

To confirm that non-plasmid bearing strains of *B. mallei* grown in minimal media expressed T6SS-1, we next examined Hcp1 production in SR1A and wild type *B. mallei* strains ATCC 23344, NCTC 3708 and NCTC 3709. As shown in Figure 3, Hcp1 was detectable in whole cell lysates prepared from cultures grown in M9G for all strains tested. In an attempt to improve the growth of the *B. mallei* strains in minimal media, we next supplemented the M9G with either casamino acids (M9CG) or tryptone (M9TG). When Hcp1 levels were assessed under these growth conditions, results demonstrated robust Hcp1 production during growth in M9CG, but much lower Hcp1 production during growth in M9TG (Figure 3). As expected, Hcp1 was virtually undetectable when the *B. mallei* strains were grown in LB4G. Taken together, these findings indicated that we had identified media conditions (M9G or M9CG) that induced Hcp1 production by *B. mallei* during *in vitro* growth.

### *B. mallei hcp1* and *virG* Transcript Levels Increase during Growth in Minimal Media

**Figure 4:**
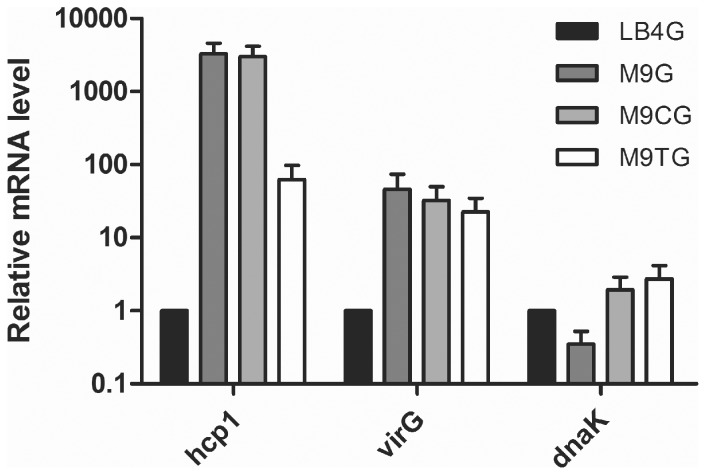
Quantitation of *B. mallei hcp1* and *virG* transcript during growth in minimal media. Transcript levels of *hcp1*, *virG* and *dnaK* were determined by qRT-PCR using gene specific primers and dual-labeled Taqman probes. RNA was harvested from *B. mallei* SR1A grown for 8 h in LB4G, M9G, M9CG or M9TG. Relative mRNA levels were calculated using the ΔΔC_T_ method and represent fold changes in comparison to growth in LB4G. All values have been normalized to the internal control, *rpoA*. Results represent the means and standard deviations of three independent experiments performed in triplicate.

Previous work from our laboratory and others has shown that *hcp1* transcript levels increase significantly in both *B. mallei* and *B. pseudomallei* when *virAG* is over-expressed *in vitro* [23], [50]. To examine T6SS-1 gene expression during growth of *B. mallei* in minimal media conditions, we harvested RNA from SR1A grown in M9G, M9CG, M9TG and LB4G, and used qRT-PCR to determine *hcp1* and *virG* transcript levels. As shown in Figure 4, in comparison to LB4G grown cultures, both M9G and M9CG grown cultures showed a >1000-fold increase in *hcp1* transcript levels. Consistent with the observations in Figure 3, M9TG grown cultures showed only a ∼62-fold increase in *hcp1* transcript when compared to growth in LB4G (Figure 4). To confirm that *virG* was expressed during growth of *B. mallei* in minimal media, we also assessed *virG* mRNA levels by qRT-PCR. As would be predicted, *virG* transcript levels were increased in M9G, M9CG and M9TG grown bacteria (45-, 32- and 22-fold up-regulation, respectively) in comparison those grown in LB4G (Figure 4). The levels of the housekeeping gene, *dnaK,* remained relatively unchanged. These findings provide further evidence that growth in minimal media induces T6SS-1 expression *in vitro*.

### *B. mallei virG* is Required for T6SS-1 Expression during Growth in Minimal Media

**Figure 5:**
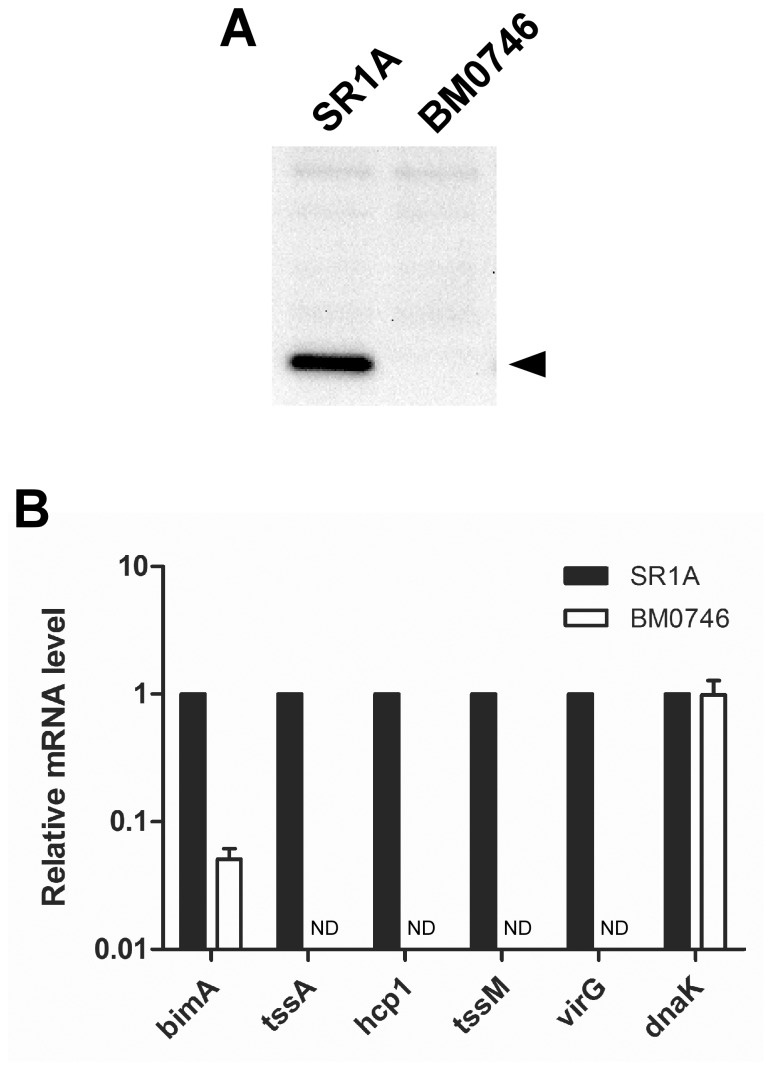
*B. mallei* T6SS-1 expression in M9CG media is VirG-dependent. (A) Whole cell lysates were prepared from overnight cultures of *B. mallei* SR1A or BM0746 (Δ*virG*) grown in M9CG and then assayed for Hcp1 production by Western immunoblotting using anti-BmHcp1 polyclonal rat serum. The protein band corresponding to Hcp1 is indicated by the black arrowhead. (B) Transcript levels of *bimA*, *tssA*, *hcp1*, virG, *tssM* and *dnaK* were determined by qRT-PCR using gene specific primers and dual-labeled Taqman probes. RNA was harvested from *B. mallei* strains grown for 8 h in M9CG. Relative mRNA levels were calculated using the ΔΔC_T_ method and represent fold changes in comparison to SR1A. All values have been normalized to the internal control, *rpoA*. Results represent the means and standard deviations of three independent experiments performed in triplicate. ND, not detected.

In a previous study, expression of *hcp1* and *tssA* in *B. pseudomallei* was shown to be dependent on VirAG during growth in macrophages, but not during growth in RPMI medium [51]. To determine if *virG* was required for T6SS-1 expression in *B. mallei* during growth in M9CG media, we constructed a *virG* deletion mutant (BM0746) and assessed Hcp1 production. As shown in Figure 5A, Western immunoblot analysis demonstrated that Hcp1 was undetectable in BM0746. We next examined *hcp1* and *tssA* mRNA levels in BM0746 in comparison to SR1A during growth in M9CG media using qRT-PCR. In agreement with the immunoblot analysis, results indicated that in the absence of VirG, *hcp1* and *tssA* transcripts were undetectable (Figure 5B). We extended these studies to examine the expression of additional VirAG regulated genes including *tssM* and *bimA*. While *tssM* transcript was undetectable in the *virG* mutant, *bimA* transcript was reduced ∼20-fold in comparison to the parent strain (Figure 5B). As expected, *dnaK* transcript levels in the *virG* mutant were comparable to the parent strain. Collectively, these findings showed that while *hcp1*, *tssA* and *tssM* expression appeared to be completely VirG-dependent, *bimA* expression was only partially VirG-dependent during growth of *B. mallei* in M9CG medium.

### *B. mallei* T6SS-1 Expression is Regulated by Divalent Metals

**Figure 6:**
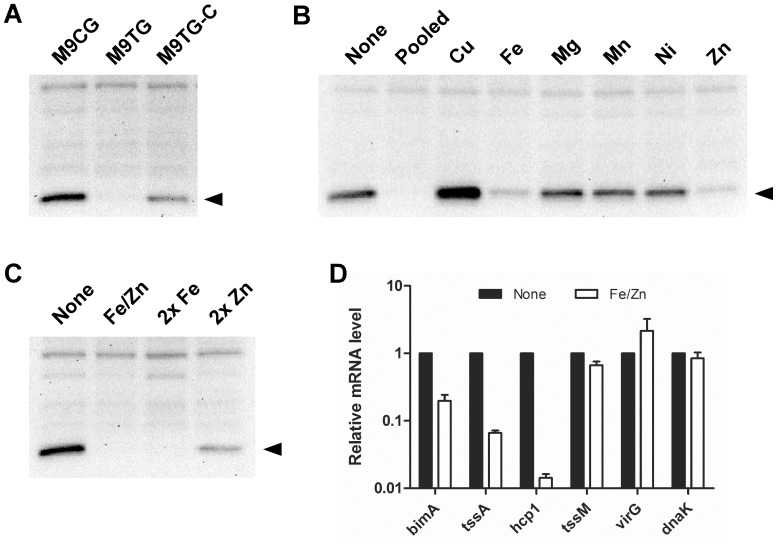
Iron and zinc inhibit *B. mallei* T6SS-1 expression. Whole cell lysates were prepared from overnight cultures of *B. mallei* SR1A grown in: (A) M9CG, M9TG or M9TG-C; (B) M9CG alone (none), M9CG supplemented with copper, iron, magnesium, manganese, nickel and zinc (pooled; 10 µM each), M9CG individually supplemented with copper, iron, magnesium, manganese, nickel or zinc (Cu, Fe, Mg, Mn, Ni or Zn; 10 µM each) or; (C) M9CG alone (none), M9CG supplemented with iron and zinc (Fe/Zn, 10 µM each), 2x iron (2x Fe, 20 µM) or 2x zinc (2x Zn, 20 µM); and then assayed for Hcp1 production by Western immunoblotting using anti-BmHcp1 polyclonal rat serum. The protein band corresponding to Hcp1 is indicated by the black arrowheads. (D) Transcript levels of *bimA*, *tssA*, *hcp1*, *virG, tssM* and *dnaK* were determined by qRT-PCR using gene specific primers and dual-labeled Taqman probes. RNA was harvested from *B. mallei* strains grown for 8 h in M9CG (none) or M9CG plus iron and zinc (Fe/Zn; 10 µM each). Relative mRNA levels were calculated using the ΔΔC_T_ method and represent fold changes in comparison to M9CG. All values have been normalized to the internal control, *rpoA*. Results represent the means and standard deviations of three independent experiments performed in triplicate.

The observation that *hcp1* was highly expressed during growth of *B. mallei* in M9CG, but at much lower levels in M9TG, prompted us to more closely compare the components of the media supplements (i.e., casamino acids and tryptone) used in this study. While both are digests/hydrolysates of casein, casamino acids preparations are largely devoid of iron in comparison to tryptone. To determine if the presence of iron or other divalent metals in tryptone suppressed Hcp1 production, *B. mallei* was grown in minimal medium supplemented with Chelex-treated tryptone (M9TG-C) and Hcp1 production was assessed. Results of Western immunoblots showed that, although not as robust as in M9CG grown *B. mallei*, Hcp1 was detectable in M9TG-C grown cultures (Figure 6A). Extending upon this observation, we next grew *B. mallei* in M9CG supplemented with various divalent metals and examined Hcp1 levels. Results demonstrated that either iron or zinc significantly inhibited Hcp1 production (Figure 6B). When *B. mallei* was grown in M9CG supplemented with both iron and zinc (10 µM), or if the iron concentration was increased 2-fold (20 µM), Hcp1 was virtually undetectable (Figure 6C). In support of these observations, qRT-PCR experiments confirmed that *hcp1*and *tssA* transcript levels decreased (∼60- and 16-fold down-regulation, respectively) during growth of *B. mallei* in M9CG containing both iron and zinc (Figure 6D). In addition, *bimA* transcript levels decreased (∼5-fold), but *virG*, *tssM* and *dnaK* transcript levels remained relatively unchanged in the presence of iron and zinc. Taken together, these findings indicated that divalent metals, particularly iron and zinc, play a role in negatively regulating T6SS-1 and *bimA* expression in *B. mallei*.

### *B. pseudomallei*, but not *B. thailandensis*, T6SS-1 is Expressed during Growth in M9CG and is Regulated by Iron and Zinc

**Figure 7:**
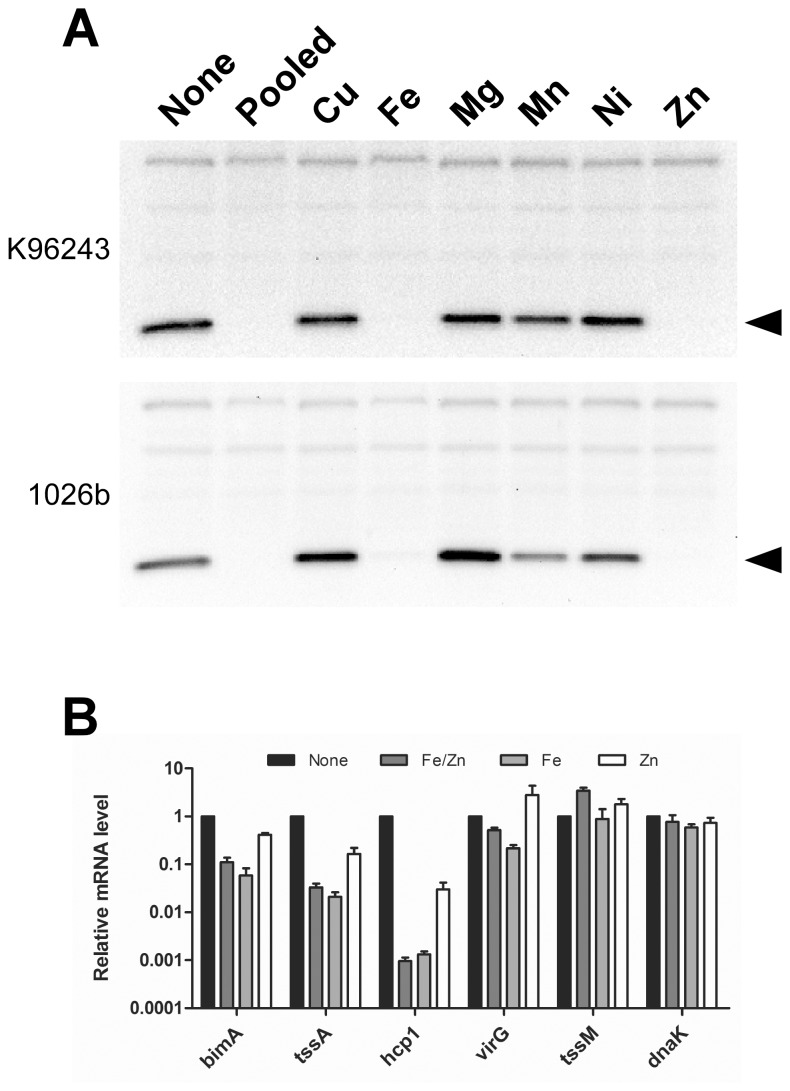
*B. pseudomallei* T6SS-1 expression is suppressed by iron and zinc. (A) Whole cell lysates were prepared from overnight cultures of *B. pseudomallei* K96243 and 1026b grown in M9CG alone (none), M9CG supplemented with copper, iron, magnesium, manganese, nickel and zinc (pooled; 10 µM each), or M9CG individually supplemented with copper, iron, magnesium, manganese, nickel or zinc (Cu, Fe, Mg, Mn, Ni or Zn; 10 µM each), and then assayed for Hcp1 production by Western immunoblotting using anti-BmHcp1 polyclonal rat serum. The protein band corresponding to Hcp1 is indicated by the black arrowheads. (B) Transcript levels of *bimA*, *tssA*, *hcp1*, *virG, tssM* and *dnaK* were determined by qRT-PCR using gene specific primers and dual-labeled Taqman probes. RNA was harvested from *B. pseudomallei* K96243 grown for 8 h in M9CG (none), M9CG plus iron and zinc (Fe/Zn; 10 µM each), M9CG plus iron (Fe; 10 µM), or M9CG plus zinc (Zn; 10 µM). Relative mRNA levels were calculated using the ΔΔC_T_ method and represent fold changes in comparison to M9CG. All values have been normalized to the internal control, *rpoA*. Results represent the means and standard deviations of three independent experiments performed in triplicate.

*B. pseudomallei* and *B. thailandensis* are closely related to *B. mallei*, and harbor homologous T6SS-1 gene clusters (BPSS1496-1511 and BTH_II0870-0855, respectively) [23]. In both species, T6SS-1 is located adjacent to *virAG* and *bimBCADE* as shown in Figure 1. Previous studies have demonstrated that similar to *B. mallei*, *B. pseudomallei* T6SS-1 expression is induced following uptake by host cells and requires VirG [50], [51], [55], [59]. As would be anticipated based on the results obtained for *B. mallei* (Figure 3), robust Hcp1 production was detected in whole cell lysates of *B. pseudomallei* harvested from cultures grown in M9CG, but not M9TG (data not shown). To determine if Hcp1 production in *B. pseudomallei* was influenced by divalent metals, strains K96243 and 1026b were grown in M9CG alone or supplemented with various cations and Hcp1 levels were assessed. Results demonstrated that either iron or zinc negatively regulated Hcp1 production in both strains (Figure 7A). Quantitation of K96243 *hcp1*and *tssA* mRNA levels supported a role for both iron and zinc in this process. *hcp1* transcript levels decreased ∼750-fold in the presence of iron and ∼33-fold in the presence of zinc (Figure 7B). Likewise, *tssA* transcript levels decreased ∼40-fold in the presence of iron and ∼6-fold in the presence of zinc. Interestingly, *bimA* transcript levels decreased ∼10–17-fold in response to iron alone or iron and zinc together, but only ∼2.5-fold in response to zinc alone. For the most part, *virG* mRNA levels remained relatively constant, however a 5-fold decrease was noted in the presence of iron alone. Both *tssM* and *dnaK* transcript levels remained relatively unchanged in the presence of iron and zinc (Figure 7B). Overall, these findings were largely consistent with those observed for *B. mallei* (Figure 6).

**Figure 8:**
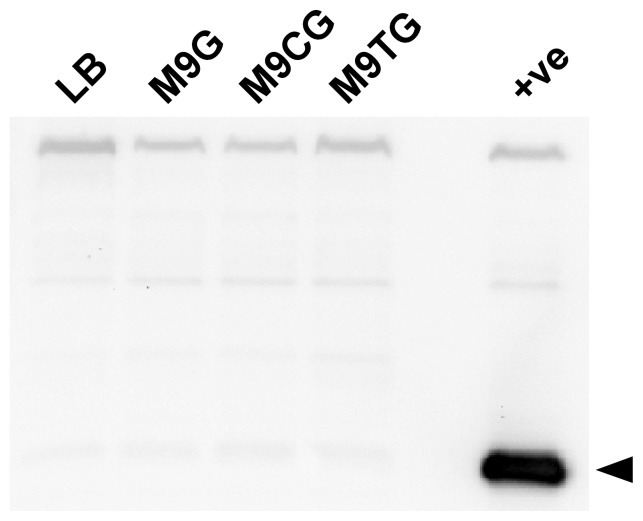
Hcp1 is not produced by *B. thailandensis* during growth in minimal media. Whole cell lysates were prepared from overnight cultures of *B. thailandensis* E264 grown in LB4G, M9G, M9CG or M9TG, and then assayed for Hcp1 production by Western immunoblotting using anti-BtHcp1 polyclonal rabbit serum. A whole cell lysate prepared from *B. thailandensis* DW503 (pBtvirAG) grown overnight in LB was used as a positive control (+ve) for Hcp1 expression. The protein band corresponding to Hcp1 is indicated by the black arrowhead.

To assess the expression of *B. thailandensis* T6SS-1, we raised anti-serum against recombinant *B. thailandensis* Hcp1 (rBtHcp1) and used it to monitor Hcp1 production during growth of the organism in LB, M9G, M9CG and M9TG. In contrast to the pathogenic species, *B. thailandensis* did not produce Hcp1 under any of the media conditions tested (Figure 8). Notably, however, Hcp1 production could be induced by expressing *virAG* on a multicopy plasmid. Based upon these observations, it appears that distinct regulatory mechanisms may govern T6SS-1 gene expression in *B. thailandensis* in comparison to *B. mallei* and *B. pseudomallei*.

## Discussion

Pathogens harboring virulence-associated T6SSs that target eukaryotic cells typically do not express these systems under routine laboratory growth conditions [17], [23], [48]–[57]. For the most part, expression of such T6SSs is tightly regulated at the transcriptional level so that they are only produced when appropriate environmental cues are present [17], [23], [48]–[57]. In keeping with this notion, T6SS-1 genes are poorly transcribed during growth of *B. mallei* and *B. pseudomallei* in rich media, but are significantly up-regulated following internalization by host cells [17], [23], [50], [51], [55]. These observations are consistent with an important role for T6SS-1 in the intracellular behavior of both *B. mallei* and *B. pseudomallei* [17], [50], [51], [55], [60]. In the present study, we describe defined minimal media conditions that activate T6SS-1 expression during *in vitro* growth of these organisms. Using Hcp1 production as an indicator of T6SS-1 expression, we found that growth of *B. mallei* or *B. pseudomallei* in M9CG media supported robust expression of this system. In addition, the up-regulation of *hcp1* transcript was confirmed to be >1000-fold by qRT-PCR. These findings are in agreement previous studies indicating that expression levels of both the T3SS_AP_ and T6SS-1 genes were generally higher when *B. pseudomallei* was cultured in RPMI 1640, a defined media commonly used for tissue culture, than in rich media formulations such as LB or DMEM [51], [59].

Although the regulation of T6SSs varies widely from species to species, some common mechanisms that govern T6SS gene expression include quorum sensing, changes in temperature and pH, and two-component regulatory systems [28], [38], [47], [48]. Regulation of T6SS-1 in *B. mallei* and *B. pseudomallei* appears to be complex and to involve several transcriptional regulators including BspR, BprP, BsaN, BprC (BMAA1517) and VirAG [23], [50], [51], [59]. In both organisms, overexpression of *virAG* results in transcription of the T6SS-1 gene cluster as well as the production and export of Hcp1 [23], [50]. To date, the specific signals sensed by VirAG that lead to T6SS-1 expression have not been elucidated. In this study, we demonstrated that *virG* is expressed during growth in M9CG media and is required for T6SS-1, *bimA* and *tssM* expression. Our results showed that *virG* transcript levels increased (∼32-fold) during growth of *B. mallei* in M9CG in comparison to LB4G. Analysis of a *B. mallei* Δ*virG* mutant confirmed a critical role for VirG in the expression of T6SS-1 genes (*tssA* and *hcp1*) and *tssM* transcription, and to a lesser extent *bimA* transcription. These findings are comparable to those from a previous study reporting that the activation of T6SS-1 in *B. pseudomallei* was completely dependent on VirAG within the intracellular environment [51]. Our results, however, are in contrast to findings in the same study indicating that deletion of *virAG* in *B. pseudomallei* had no effect on T6SS-1 gene expression when bacteria were grown in RPMI medium. This discrepancy may be due to differences in the levels of T6SS-1 gene expression in RPMI in comparison to M9CG. It is conceivable that VirAG regulated genes may be transcribed at much higher levels during growth of the organisms in M9CG than in RPMI. Further studies will be necessary to address this issue. Taken together, however, these findings suggest that growth of *B. mallei* and *B. pseudomallei* in M9CG provides a signal(s) sensed by VirAG which induces its expression *in vitro*.

Several recent studies have identified a variety of environmental cues that influence T6SS gene expression including the extracellular concentrations of iron, phosphate and magnesium [61]–[65]. In *Edwardsiella* species, T3SS and T6SS genes have been shown to be activated under conditions that mimic those of a phagosome (i.e., low pH and limited phosphate) and repressed in the presence of high iron [63], [64]. Similarly, in *Pseudomonas aeruginosa* and enteroaggregative *Escherichia coli*, expression of the H2-T6SS and *sci1* T6SS gene clusters, respectively, is repressed in the presence of iron [61], [65]. During initial growth media optimization experiments with *B. mallei*, we made the observation that when M9G media was supplemented with tryptone (iron-replete) rather than casamino acids (iron-deplete), Hcp1 production was repressed. Extending upon this finding, we showed that iron and zinc negatively regulate Hcp1 production in both *B. mallei* and *B. pseudomallei*. Analysis of mRNA levels revealed that transcription of *tssA*, *hcp1* and *bimA* was down-regulated in the presence of iron/zinc, but that *tssM* transcript was unchanged, indicating that not all VirAG regulated genes are metal regulated. While *virG* transcript levels remained relatively constant in both organisms in the presence of iron/zinc, *virG* transcript decreased ∼5-fold *B. pseudomallei* in the presence of iron alone. The reason for this result is currently unclear. Additional experiments will be necessary to investigate this phenomenon as well as to determine the levels and activation state of VirG under these growth conditions.

Based on the results obtained herein, it appears that while VirAG positively regulates T6SS-1 expression, iron and zinc negatively regulate the expression of this important secretion system. Since vertebrate hosts sequester iron as a protective mechanism against bacterial infections, and pathogens often sense this iron depletion as a signal to express virulence genes, our findings are in keeping with previous studies demonstrating that T6SS-1 is expressed within host environments [17], [51], [55]. In addition, it has been shown that the Natural resistance-associated macrophage protein 1 (Nramp1), a multi-specific symporter that facilitates the efflux of divalent cations from host phagosomes, restricts microbial access to essential metals within this sub-cellular compartment [66]. This concept is consistent with our previous observation that *B. mallei* T6SS-1 is expressed within phagolysosomal compartments and with the findings in the current study demonstrating that iron and zinc limiting conditions induce expression of genes encoding this critical virulence factor [17].

The ferric uptake regulator protein Fur has been shown to be involved in the regulation of expression of the *sci1* T6SS in enteroaggregative *E. coli*, the H2-T6SS in *P. aeruginosa* and, a T6SS in *Edwardsiella tarda* [61], [65]. In both *E. coli* and *P. aeruginosa*, Fur negatively regulates T6SS expression in the presence of iron by binding to Fur boxes in the promoter regions of the gene clusters encoding these systems. The situation in *E. tarda* appears to be more complex, and it has recently been reported that in addition to directly suppressing T6SS, Fur also acts indirectly through unidentified downstream transcriptional regulators that interact with components of the PhoB regulon [63]. Two Fur homologs (BPSL2943/BMA2458 and BPSL0825/BMA0329) have been identified in the genomes of *B. mallei* ATCC 23344 and *B. pseudomallei* K96243 [67]. The potential role of these Fur proteins in the regulation of T6SS-1 has not yet been explored. It remains to be determined whether or not Fur boxes exist upstream of T6SS-1 and if either of the putative *B. mallei*/*B. pseudomallei* Fur proteins play a role in regulating T6SS-1 gene expression.

*B. thailandensis* produces a number of homologs of known virulence determinants that are expressed by *B. mallei* and *B. pseudomallei* and is commonly used as a model system to study specific aspects related to the pathogenesis of glanders and melioidosis [16]. In addition, *B. thailandensis* can invade and replicate within host cells and exhibits a similar intracellular lifestyle as the pathogens [16], [68]. Previous reports indicate that the phenotypes associated with *B. thailandensis* T6SS-1 mutants are similar those displayed by *B. mallei* and *B. pseudomallei* T6SS-1 mutants, i.e. these strains do not stimulate MNGC formation and are attenuated in a mouse model of acute pneumonic *B. thailandensis* infection [60], [69]. In line with these observations, overexpression of VirAG in *B. thailandensis* activates T6SS-1 expression and results in Hcp1 production. Curiously, however, in contrast to *B. mallei* and *B. pseudomallei*, growth of *B. thailandensis* in M9G and M9CG media did not induce Hcp1 production. These findings suggest that the regulatory mechanisms controlling T6SS-1 expression in *B. thailandensis* may differ from those used by the pathogens. Although VirAG plays a role in regulating T6SS-1, it appears that under the conditions tested, divalent metals do not. Whether *B. thailandensis* is an appropriate surrogate for studying the regulation of this virulence-associated T6SS requires additional investigation. It is tempting to speculate that differences in the regulatory mechanisms governing virulence factor expression in these three *Burkholderia* species may account, in part, for the differences in their pathogenicity.

In summary, we have identified *in vitro* growth conditions that induce Hcp1 production in *B. mallei* and *B. pseudomallei*. We demonstrated that *virG* is expressed under these conditions and is required for T6SS-1 gene expression. In addition, we showed that iron and zinc down-regulate *tssA*, *hcp1* and *bimA* expression. Other genes (i.e., *tssM*) under the control of VirAG did not appear to be as tightly regulated by these metals. Collectively, our findings indicate that in addition to being positively regulated by VirAG, *B. mallei* and *B. pseudomallei* T6SS-1 gene expression is negatively regulated by iron and zinc. The regulation of T6SS-1 appears to be complex and may involve VirG-independent mechanisms that have not yet been identified. Nevertheless, the media conditions described in this study will facilitate additional investigations aimed at characterizing the functions of these important intracellular virulence factors at a molecular level and will be useful for identifying *in vivo* expressed antigens for use in the development of glanders and melioidosis diagnostics and vaccines.
